# Supplementary material for: Examining Association of Personality Characteristics and Neuropsychiatric Symptoms in Post-COVID Syndrome
Source: Brain Sci. 2022 Feb 14;12(2):265. doi: 10.3390/brainsci12020265 (PMC8870488; doi:10.3390/brainsci12020265)
Supplement: Supplementary file 1 [file brainsci-12-00265-s001.zip › Supplementary Table S1.pdf]

Table S1. Neuropsychological tests.

Corsi block-tapping test
Symbol Digit Modalities Test
Boston Naming Test
Judgment of Line Orientation
Rey-Osterrieth Complex Figure (copy and recall at 3 and 30 minutes, and recognition)
Free and Cued Selective Reminding Test
Verbal fluencies (animals and words beginning with "p" and "m"; 1 minute per category)
Stroop Color and Word Test
Visual Object and Space Perception Battery (object decision, progressive silhouettes, number location, position discrimination)
Trail Making Test (S1 form) (VTS)
Figural Memory Test (S11 form) (VTS)
Tower of London (TOL-F, S1 form) (VTS)
Response inhibition (S13 form) (VTS)
N-Back Verbal Test (S1 form) (VTS)
Cognitrone (S11 form) (VTS)
Reaction Test (RT, S3 form) (VTS)
Determination Test (DT, S1 form) (VTS)
WAF battery (S1 form) (VTS)

VTS: Vienna Test System®; WAF: Perception and attention functions test.
