# Supplementary figures and images for: Examining Association of Personality Characteristics and Neuropsychiatric Symptoms in Post-COVID Syndrome
Source: Brain Sci. 2022 Feb 14;12(2):265. doi: 10.3390/brainsci12020265 (PMC8870488; doi:10.3390/brainsci12020265)

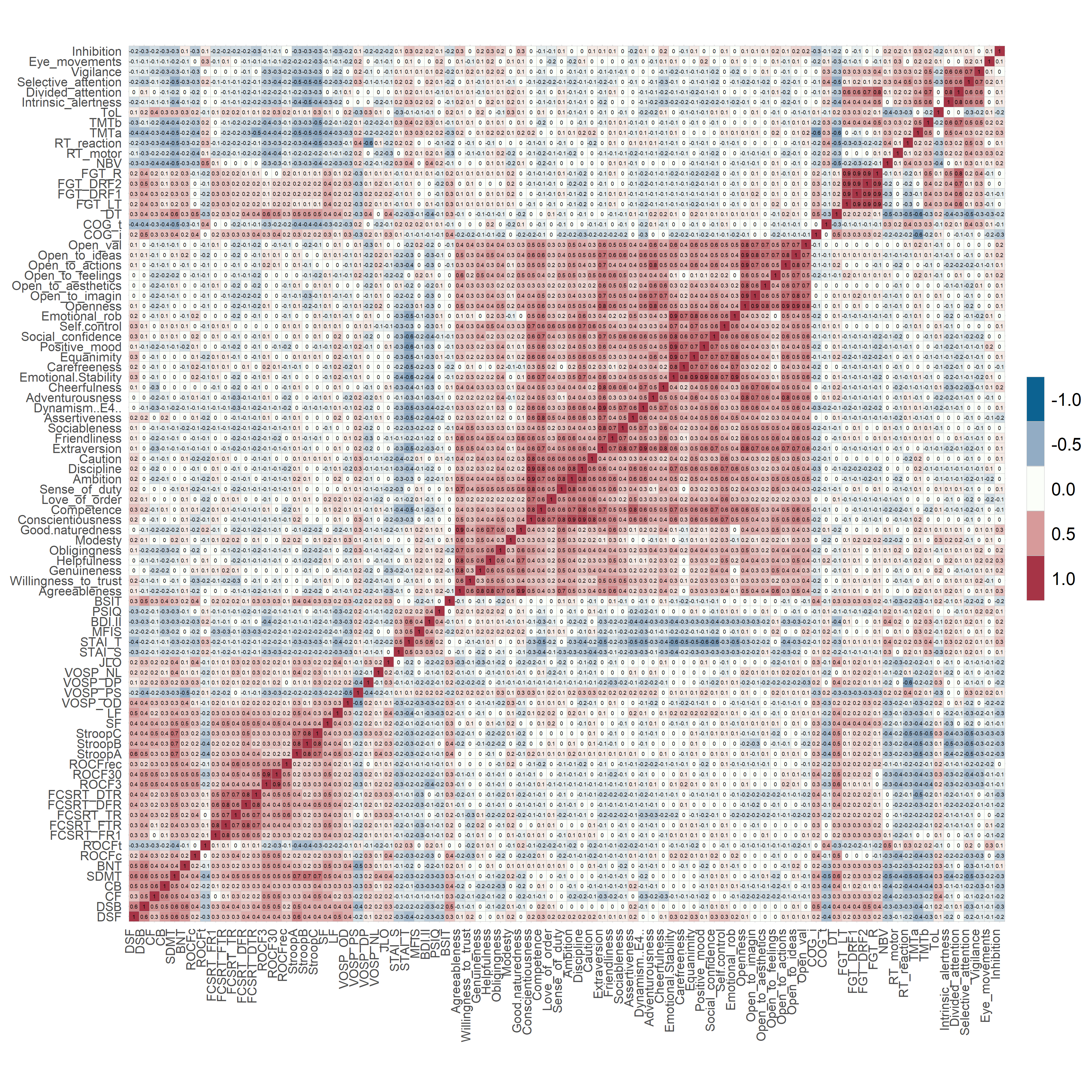

Supplement: Supplementary file 1 [file brainsci-12-00265-s001.zip › Supplementary Figure S1.tiff]
